# Analysis of Membrane Transport Equations for Reverse Electrodialysis (RED) Using Irreversible Thermodynamics

**DOI:** 10.3390/ijms21176325

**Published:** 2020-08-31

**Authors:** Wojciech Kujawski, Andriy Yaroshchuk, Emiliy Zholkovskiy, Izabela Koter, Stanislaw Koter

**Affiliations:** 1 Faculty of Chemistry, Nicolaus Copernicus University in Toruń, 87-100 Toruń, Poland; ikoter@umk.pl; 2 MEPhI, National Research Nuclear University, 115409 Moscow, Russia; 3 ICREA & Polytechnic University of Catalonia—Barcelona Tech, 08034 Barcelona, Spain; andriy.yaroshchuk@upc.edu; 4 Institute of Bio-Colloid Chemistry, National Academy of Sciences of Ukraine, 03680 Kyiv-142, Ukraine; emiliy@ualberta.ca

**Keywords:** reverse electrodialysis, irreversible thermodynamics, power density, Nafion 120 membrane

## Abstract

Reverse electrodialysis (RED) is an electro-membrane process for the conversion of mixing energy into electricity. One important problem researchers’ face when modeling the RED process is the choice of the proper membrane transport equations. In this study, using experimental data that describe the membrane Nafion 120 in contact with NaCl aqueous solutions, the linear transport equation of irreversible thermodynamics was applied to calculate the power density of the RED system. Various simplifying assumptions about transport equation (i.e., four-, three-, and two-coefficients approaches) are proposed and discussed. We found that the two-coefficients approach, using the membrane conductivity and the apparent transport number of ions, describes the power density with good accuracy. In addition, the influence of the membrane thickness and the concentration polarization on the power density is also demonstrated.

## 1. Introduction

Global population growth contributes to the escalation of energy demand for households, transportation, and industry. The use of fossil fuels results in various environmental threats. Therefore, the search is increasingly widespread for alternative methods of energy conversion, e.g., osmotic into electrical [1] and mechanical energy (so called “osmotic pump” [2,3], or the conversion of electrokinetic energy [4]. Reverse electrodialysis (RED) is an electro-membrane process for the conversion of mixing energy into electricity. The concept of RED was invented in 1950 [5,6]; however, it was not heavily investigated until the 21st century, when this technique regained the attention of researchers looking for novel, unconventional, and renewable energy sources [7,8,9].

One of the important RED issues is the cost-effectiveness of the process. Turek and Bandura [10] suggested that energy conversion using RED is not profitable; however, Giacalone et al. [11] presented a much more optimistic perspective. There are a number of parameters that influence the efficiency of RED, like ionic shortcut currents [12], type of electrodes [13], kind of membrane [14], membrane thickness [15], fouling issues [16,17], efficiency of solutions mixing in the stack [18,19], and geometry of spacers [20].

In this section, we present a description of membrane transport in RED. In the literature discussing the modeling of RED, apart from very simplified membrane transport (i.e., considering only the transport numbers of ions [21]), usually the Nernst–Planck equation or its transformations are used [22,23,24,25,26,27,28]. In some research, the volume flow caused by the osmotic and electroosmotic transport of water through the membrane was considered [25,29]. Whereas in other research, only osmotic transport was considered [22,24]. In other studies, no volume transport was anticipated [27]. The problem of membrane transport is probably neglected because of the many other important aspects of RED. A general (i.e., not exactly related to RED) review of transport through ion-exchange membranes can be found elsewhere [30].

The Nernst–Planck equation is a simplified version of the linear transport equation of irreversible thermodynamics. The transport equation is comprised of six transport coefficients for the system membrane in contact with a single electrolyte aqueous solution, under the condition that the Onsager symmetry of the coefficients is fulfilled [31]. The determination of all these coefficients requires extensive work especially because their concentration dependence should be considered [32,33,34,35]. Consequently, the practical use of the full transport equation is limited.

Therefore, the aim of this work was to discuss which of the simplifications of the irreversible thermodynamics transport equation (ITE) is useful for calculating the power density in RED. The following three approaches based on four, three, and two coefficients (to be determined experimentally) were analyzed and discussed in detail:

(1) the four-coefficients approach—the pressure difference in the full set of ITE is neglected,
(2) the three-coefficients approach—the coupling of ion flows with the gradient of chemical potential of water is neglected,
(3) the two-coefficients approach (i.e., Nernst–Planck equation)—the ion flux is a function of the gradient of its electrochemical potential only.

The results of these simplifications (i.e., values of power density vs. electric current density) are compared with the values obtained from the full set of ITE. The transport coefficients used in these latter equations were determined for the cation-exchange membrane Nafion 120 in contact with NaCl solutions [34,35] in a wide range of concentrations (0.05–3.7 M). To consider the concentration dependence, the differential form of the transport equation was used. This method was applied for the first time by McCallum and Meares to describe transport through an ion-exchange membrane [36]. The Nafion 120 membrane is relatively thick (0.3 mm); therefore, the transport coefficients are rescaled to smaller thickness values to demonstrate the influence of that parameter on the power density.

## 2. Theory

**Figure 1:**
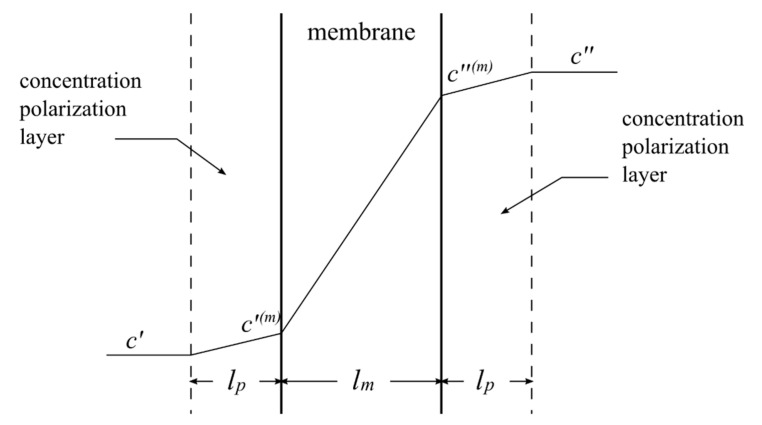
The system: ion-exchange membrane with adjacent boundary diffusion layers. l_p_—thickness of concentration polarization layer; l_m_—thickness of the membrane; c′, c″—concentrations of ions in the bulk solution; c′^(m)^, c″^(m)^—concentrations of ions at the membrane surface.

In the following, one basic part of the RED module is considered, i.e., one ion-exchange membrane with adjacent concentration polarization layers (Figure 1). The steady-state conditions in the system are assumed, i.e., the fluxes of ions in the membrane and both polarization layers are the same.

### 2.1. Transport Equations of Irreversible Thermodynamics (ITE)

The starting point for the ITE approach to isothermal conditions is the dissipation function, Φ, the product of entropy production, and temperature, which can be expressed as the sum of driving forces, *X_i_*, and coupled with fluxes, *J_i_*:

$$\Phi =T\sigma =\sum_{i}J_{i}X_{i}>0.$$

For discontinuous systems, the forces are represented by the differences in chemical potentials of transported species. In continuous systems, the negative values of gradients of these potentials are used. 

According to irreversible thermodynamics, it is assumed that fluxes and forces are linearly related:

(1) $$J_{i}=L_{i1}X_{1}+L_{i2}X_{2}+\ldots$$

and the Onsager reciprocity relation *L_ik_* = *L_ki_* holds if the forces are not too high.

One can choose many sets of fluxes and forces; all of them have to fulfill the condition of the entropy production invariance:

(2) $$\Phi =\sum_{i}J_{i}^{\prime }X_{i}^{\prime }=\sum_{i}J_{i}^{\prime \prime }X_{i}^{\prime \prime }.$$

From this condition, we obtain various forms of the fluxes and forces, among which is the set of practical equations useful for experimental determination of transport coefficients [37]:

(3) $$\begin{array}{cc} J_{1}/\nu_{1}= & L_{\pi \pi }\Delta \pi /{\tilde{c}}_{s}+L_{\pi p}(\Delta p-\Delta \pi )+L_{\pi E}\Delta E\\ J_{v}= & L_{p\pi }\Delta \pi /{\tilde{c}}_{s}+L_{pp}(\Delta p-\Delta \pi )+L_{pE}\Delta E\\ I= & L_{E\pi }\Delta \pi /{\tilde{c}}_{s}+L_{Ep}(\Delta p-\Delta \pi )+L_{EE}\Delta E \end{array}$$

where *J*_1_ is the flux of ions 1 (cations), *ν*_1_ is the number of ions 1 in the electrolyte molecule, *s* is volume flux, *I* is electric current density, Δ*p* is the pressure difference, Δ*π* is the osmotic pressure difference, ${\tilde{c}}_{s}$ is the mean concentration of electrolyte solutions contacting the membrane (*c_s_*′, *c_s_*″), and ${\tilde{L}}_{\alpha \beta }$ is the mean transport coefficient for that concentration range. Δ*E* is the electric potential difference measured with electrodes reversible to anions (2):

(4) $$\Delta E=\Delta {\tilde{\mu }}_{2}/z_{2}F=\Delta \varphi +\frac{RT}{z_{2}F}\Delta \ln a_{2}.$$

The power density is calculated from the electric potential difference Δ*φ*, determined from Equation (4) assuming that $\Delta \ln a_{2}\approx \Delta \ln a_{\pm }$.

The determination of all *L_αβ_* coefficients is a laborious task. In Table 1, we list the experiments we performed to determine *L_αβ_* in Equation (3).

Having these coefficients determined for the vanishingly small concentration differences (Δ*π*, Δ*μ_s_* → 0) allowed us to assume the Onsager reciprocity relation (*L_αβ_* = *L_βα_*) and determine the concentration dependence of six *L_αβ_* (*α*, *β* = *π*, *p*, *E*) [34,35]. The method of such determination was described in detail elsewhere [32]. The finite concentration differences (*L_αβ_*) in Equation (3) are not symmetric because of their strong concentration dependence [32,38].

Calculating the ion flux, volume flux, and electric potential difference, considering the concentration dependence of *L_αβ_* is the most exact approach. To calculate these quantities for the assumed conditions (concentrations on both sides of membrane, electric current density, membrane thickness), we applied the method proposed by McCallum and Meares [36]. According to this method, the membrane is divided into *n*_m_ slices and for each slice the transport is given by the set of Equation (3) with local variables (electrolyte 1:1):

(5) $$\begin{array}{cc} J_{1}= & (L_{\pi \pi }({\tilde{c}}_{s})\Delta \pi /{\tilde{c}}_{s}+L_{\pi p}({\tilde{c}}_{s})(\Delta p-\Delta \pi )+L_{\pi E}({\tilde{c}}_{s})\Delta E)l_{m}/w_{sl}\\ J_{v}= & (L_{p\pi }({\tilde{c}}_{s})\Delta \pi /{\tilde{c}}_{s}+L_{pp}({\tilde{c}}_{s})(\Delta p-\Delta \pi )+L_{pE}({\tilde{c}}_{s})\Delta E)l_{m}/w_{sl}\\ I= & (L_{E\pi }({\tilde{c}}_{s})\Delta \pi /{\tilde{c}}_{s}+L_{Ep}({\tilde{c}}_{s})(\Delta p-\Delta \pi )+L_{EE}({\tilde{c}}_{s})\Delta E)l_{m}/w_{sl} \end{array}$$

where:

$$\Delta Y=Y_{k-1}-Y_{k},\;Y=\pi ,\;p,\;E$$

$${\tilde{c}}_{s}=\left(c_{s,k-1}+c_{s,k}\right)/2$$

where *l_m_* is the thickness of the real membrane for which the coefficients *L_αβ_* = *f*(*c_s_*) were determined and *w_sl_* is the width of slice equal to the assumed membrane thickness (*l_m_*_,assumed_) divided by the number of slices (*w_sl_* = *l_m_*_,assumed_/*n*_m_). The concentration difference is expressed as Δπ and the formula $\Delta \pi =\partial \pi /\partial c_{s}(\tilde{c})\left(c_{s,k-1}-c_{s,k}\right)$.

The fluxes across each slice are the same, assuming the stationary state in the considered system. Therefore, for *n*_m_ slices, we have 3*n* equations and 3*n*_m_ unknowns: *c_s,k_*, *p_k_*, *E_k_*, *k* = 1, …, *n* − 1, *J*_1_, and *J_v_*, which we solve for given values of parameters (electric current, concentration, pressure on both sides of membrane, and the assumed membrane thickness). All of these refer to the hypothetical (virtual) solution being in equilibrium with the membrane at coordinate *x* = *kw_sl_*, *k* = 1, …, *n* − 1; such a solution is defined by equality of chemical potentials of the transported species in that solution and in the membrane at a given *x*.

The concentration polarization layers adjacent to membrane surfaces were treated in a similar way as the membrane. The experimental data for NaCl solutions were taken from [39]. The used equations are provided in Appendix A.

A comment should be made on the extended Nernst–Planck equation, frequently used in the literature for describing the membrane transport of ions. Written in terms of hypothetical solution parameters, it takes the following form:

$$J_{i}=\frac{P_{i}c_{i}}{RT}\left(-\frac{d{\tilde{\mu }}_{i}}{dx}\right)+c_{i}K_{p,i}\alpha_{i}J_{v}$$

where *K_p,i_* is the partition coefficient of ion *I* and *α_i_* is the convective coupling coefficient [40,41]. There are 2 coefficients per ion (*K_p,i_α_i_* is treated as one coefficient); thus, for two ions and *J_v_* described by 3 coefficients (*L_pπ_*, *L_pp_*, *L_pE_*), there are 7 coefficients—more than in the set of Equation (3) assuming the symmetry of coefficients. For this reason, the extended Nernst–Planck equation will not be discussed here.

### 2.2. The Four-Coefficients Approach

In this case, the pressure difference across each slice is assumed to be zero. Although on both sides of membrane the pressure is the same, it may change across the membrane to maintain the volume flux across each slice the same. As the number of unknowns is reduced, the equation for *J_v_* in Equation (5) can be neglected (*J*_1_ is needed in the concentration polarization layers). Thus Equation (5) is reduced to:

(6) $$\begin{array}{cc} J_{1}= & \left({L^{\prime }}_{\pi \pi }({\tilde{c}}_{s})\Delta \pi +L_{\pi E}({\tilde{c}}_{s})\Delta E\right)l_{m}/w_{sl}\\ I= & \left({L^{\prime }}_{E\pi }({\tilde{c}}_{s})\Delta \pi +L_{EE}({\tilde{c}}_{s})\Delta E\right)l_{m}/w_{sl} \end{array}$$

where ${L^{\prime }}_{\pi \pi }({\tilde{c}}_{s})=L_{\pi \pi }({\tilde{c}}_{s})/{\tilde{c}}_{s}-L_{\pi p}({\tilde{c}}_{s})$, ${L^{\prime }}_{E\pi }({\tilde{c}}_{s})=L_{E\pi }({\tilde{c}}_{s})/{\tilde{c}}_{s}-L_{Ep}({\tilde{c}}_{s})$.

The four coefficients (*L*′*_ππ_*, *L_πE_*, *L*′*_Eπ_*, *L_EE_*) can be determined from *P_s,dif_*, ${\bar{t}}_{1}$, *k_m_*, and ${\bar{t}}_{1,app}$—the Electromotive Force (EMF) measurements. However, practically, it is better to determine the electroosmotic coefficient *W* (Table 1) and to calculate ${\bar{t}}_{1}$ from *W* and ${\bar{t}}_{1,app}$.

### 2.3. The Three-Coefficients Approach

In the equation of ion transport, the coupling with water, i.e., the term *L_i_*_0_*X*_0_ is neglected. Thus, Equation (1) with *L_ii_* replaced by *P_i_c_i_*/*RT* reduces to:

(7) $$\begin{array}{l} J_{1}=\frac{P_{1}c_{1}}{RT}X_{1}+L_{12}X_{2}\\ J_{2}=L_{21}X_{1}+\frac{P_{2}c_{2}}{RT}X_{2} \end{array}$$

where *X_i_* is:

(8) $$X_{i}\equiv -\frac{d{\tilde{\mu }}_{i}}{dx}=-\left(RT\frac{d\ln a_{i}}{dx}+z_{i}F\frac{d\phi }{dx}\right).$$

We assume that *L*_21_ = *L*_12_; therefore, there are 3 parameters that can be obtained from (1) specific conductivity, *k*_m_, (2) electrolyte permeability *P_s_*, and (3) the apparent transport number, ${\bar{t}}_{1,app}$. Instead of ${\bar{t}}_{1,app}$, we could choose the real transport number of cation, ${\bar{t}}_{1}$; however, because ${\bar{t}}_{1,app}$ includes the electroosmotic number of water and is much easier to determine than ${\bar{t}}_{1}$, we choose ${\bar{t}}_{1,app}$. Notably, ${\bar{t}}_{1,app}$ and ${\bar{t}}_{1}$ obtained from Equation (7) are the same because the flux of water is neglected here (see formulae for ${\bar{t}}_{1}$ and ${\bar{t}}_{1,app}$ in Table 1). The EMF measurements allow for the determination of the apparent transport number of ion 1. Using Equation (7), we obtain the following set of practical equations (for the 1:1 electrolyte):

(9) $$\begin{array}{cc} J_{1}= & \left(P_{1}({\tilde{c}}_{s})\left(1-{\tilde{c}}_{s}{\bar{v}}_{s}\right)\Delta \pi /RT+\left({\bar{t}}_{1,app}\kappa_{m}/F\right)\Delta E\right)/w_{sl}\\ I= & \left(\left({\bar{t}}_{1,app}\kappa_{m}/F\right)\left(1-{\tilde{c}}_{s}{\bar{v}}_{s}\right)\Delta \pi /{\tilde{c}}_{s}+\kappa_{m}\Delta E\right)/w_{sl} \end{array}$$

where *P*_1_ is given by:

$$P_{1}=P_{s}+RT\kappa_{m}{\left({\bar{t}}_{1,app}/F\right)}^{2}/c_{s},$$

and *P_s_* by:

$$P_{s}\equiv \frac{RT}{\left(1-c_{s}{\bar{v}}_{s}\right)}{\left(\frac{J_{1}}{-d\pi /dx}\right)}_{I=0}.$$

### 2.4. The Two-Coefficients Approach

In this approach, we assume that the flux of ion *I* depends only on the gradient of electrochemical potential of that ion:

(10) $$J_{i}=L_{ii}X_{i}=-\frac{P_{i}c_{i}}{RT}\frac{d{\tilde{\mu }}_{i}}{dx}\;i=1,\;2.$$

The following practical equations are derived from Equation (10):

(11) $$\begin{array}{cc} J_{1}= & P_{1}({\tilde{c}}_{s})\left(\left(1-{\tilde{c}}_{s}{\bar{v}}_{s}\right)\Delta \pi +{\tilde{c}}_{s}F\Delta E\right)/(RTw_{sl})\\ I= & P_{1}({\tilde{c}}_{s})\left(F\left(1-{\tilde{c}}_{s}{\bar{v}}_{s}\right)\Delta \pi +F^{2}{\tilde{c}}_{s}\left(1+P_{2}({\tilde{c}}_{s})/P_{1}({\tilde{c}}_{s})\right)\Delta E\right)/\left(RTw_{sl}\right) \end{array}$$

There are 2 transport coefficients only, *P*_1_ and *P*_2_, with four experimentally available parameters: (1) specific conductivity *k_m_*, (2) transport number of cation ${\bar{t}}_{1}$, (3) electrolyte permeability *P_s_*, and (4) ${\bar{t}}_{1,app}$. The relation between these quantities and *P*_1_, *P*_2_ is as follows (assuming 1:1 electrolyte, and denoting ion 1 as a cation):

(12) $$\kappa_{m}\equiv {\left(\frac{I}{-dE/dx}\right)}_{c}=\frac{F^{2}}{RT}c_{s}\left(P_{1}+P_{2}\right),$$

(13) $${\bar{t}}_{1}\equiv {\left(\frac{FJ_{1}}{I}\right)}_{c}=\frac{P_{1}}{P_{1}+P_{2}},$$

(14) $$P_{s}\equiv \frac{RT}{\left(1-c_{s}{\bar{v}}_{s}\right)}{\left(\frac{J_{1}}{-d\pi /dx}\right)}_{I=0}=\frac{P_{1}P_{2}}{P_{1}+P_{2}},$$

and

(15) $${\bar{t}}_{1,app}={\left(\frac{d{\tilde{\mu }}_{2}}{d\mu_{s}}\right)}_{I=0}=-F{\left(\frac{dE}{d\mu_{s}}\right)}_{I=0}=\frac{P_{1}}{P_{1}+P_{2}}.$$

Similar to the 3-coefficients approach, the expressions for ${\bar{t}}_{1}$ and ${\bar{t}}_{1,app}$ are the same because the water transport is also neglected.

The determination of ${\bar{t}}_{1,app}$ by the EMF method is much easier and therefore much more popular than the determination of the real transport number, ${\bar{t}}_{1}$. We chose the following pairs of experiments to determine *P*_1_ and *P*_2_: 

(1) *k_m_*, ${\bar{t}}_{1,app}$, (2) *k_m_*, *P_s_*, (3) ${\bar{t}}_{1,app}$, *P_s_*.

Additionally, contrary to ${\bar{t}}_{1}$, the value of ${\bar{t}}_{1,app}$ includes the electroosmotic transport of water; thus, to some extent, the transport of water molecules is considered.

## 3. Results and Discussion

As mentioned above, in this work, only one membrane (cation-exchange membrane) with adjacent concentration polarization layers is discussed.

**Figure 2:**
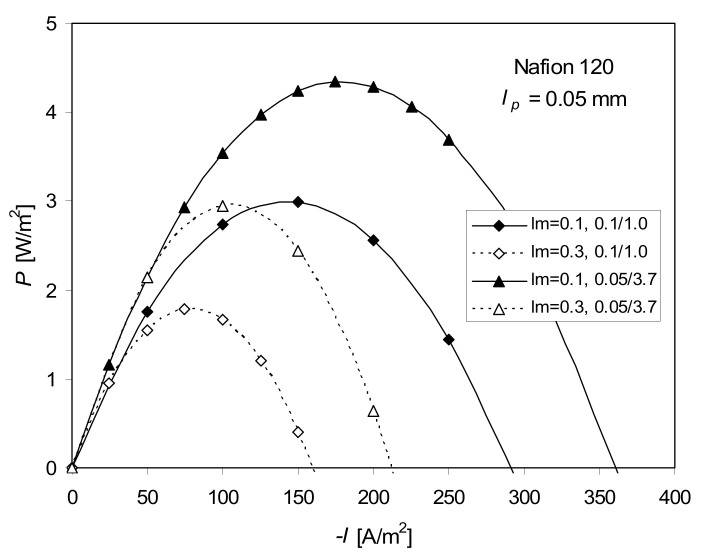
Electric power vs. −*I* calculated using Equation (5) (full ITE) for the real thickness of Nafion 120 equal to *l_m_* = 0.3 mm, and the assumed value *l_m_* = 0.1 mm, for 0.1–1.0 M and 0.05–3.7 M NaCl, *T* = 298 K. *l_p_*—thickness of the concentration polarization layer, *l_m_*—thickness of the membrane.

In Figure 2, a typical dependence of the power density *P* on the current density *I*, calculated as *P* = −*I*Δ*φ*, based on the full transport equation (Equation (5)), is shown for two concentration differences (0.1–1.0 M and 0.05–3.7 M) and two values of membrane thickness (0.1 and 0.3 mm). Two common trends were observed: the higher the concentrations difference, the higher the value of *P*; the lower the membrane thickness, the higher the value of *P*.

The four-coefficients approach, neglecting the pressure changes (Equation (6)), yields similar results. For 0.1–1.0 M NaCl, the difference in *P*_max_ is below 1% irrespective of the membrane thickness. For higher concentration differences (0.05–3.7 M), this difference increases to ca. 5–6%.

**Figure 3:**
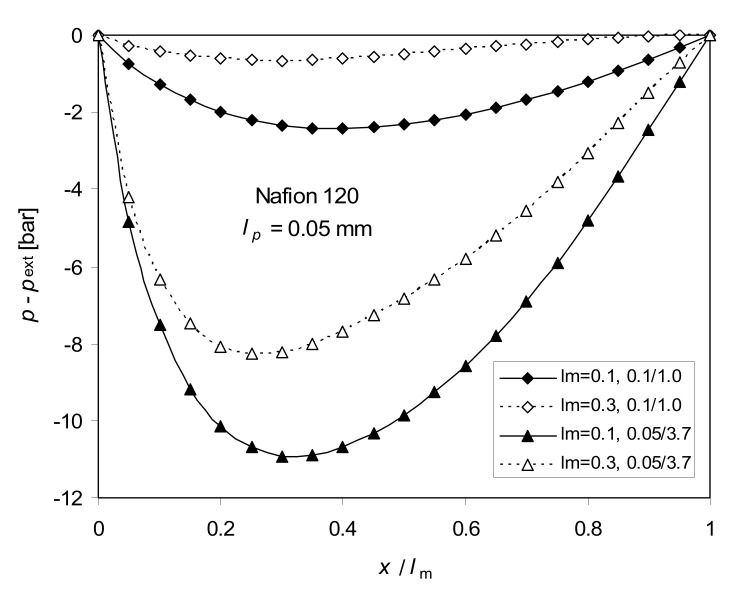
Pressure profile across the membrane calculated from Equation (5) (full ITE) for *l_m_* = 0.3 mm and *l_m_* = 0.1 mm, for 0.1–1.0 M and 0.05–3.7 M NaCl, *T* = 298 K. Thickness of the concentration polarization layer *l_p_* = 0.05 mm. Thickness of the membrane—*l_m_*.

**Figure 4:**
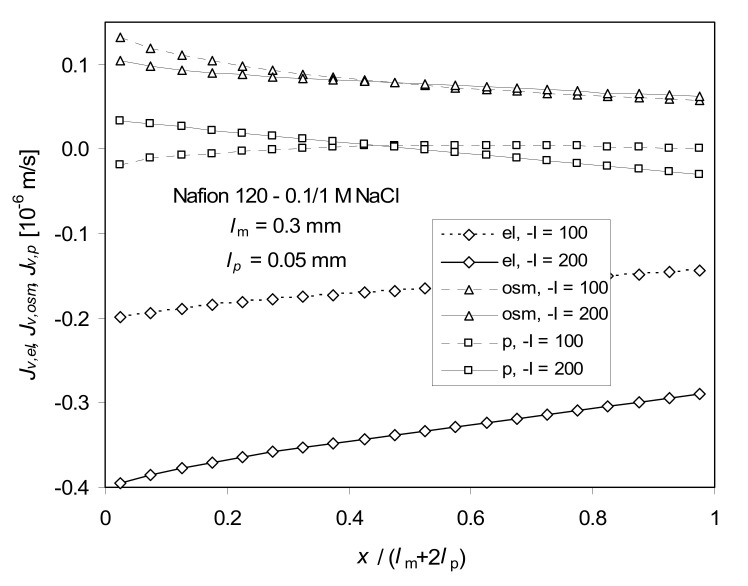
Profiles of electroosmotic (el), osmotic (osm), and pressure-driven (p), components of *J_v_* calculated from Equation (5) (full ITE) for the current density –*I* = 100 and 200 A/m^2^; one can notice a dominant role of electroosmotic contribution to *J_v_* at higher current density.

Changes of pressure in the hypothetical/virtual solution affect the ion fluxes. Some examples of pressure variations are shown in Figure 3. The negative values of pressure indicated that there must have been an underpressure inside the membrane needed to keep the volume flow constant. Similar results were obtained by McCallum and Meares [36]. They suggested that the membrane thickness could decrease in that case. At higher *I* values, the pressure becomes positive. This can be explained by different changes in the profiles of electroosmotic and osmotic parts of *J_v_* across the membrane caused by the change of concentration profiles (Figure 4). To keep *J_v_* constant, the pressure part of *J_v_* should change accordingly, which demands various profiles of pressure. Assuming the constant pressure value (the four-coefficients approach), the volume flux across the membrane is not constant—it can deviate from the actual steady state value even several times, which is unrealistic.

**Figure 5:**
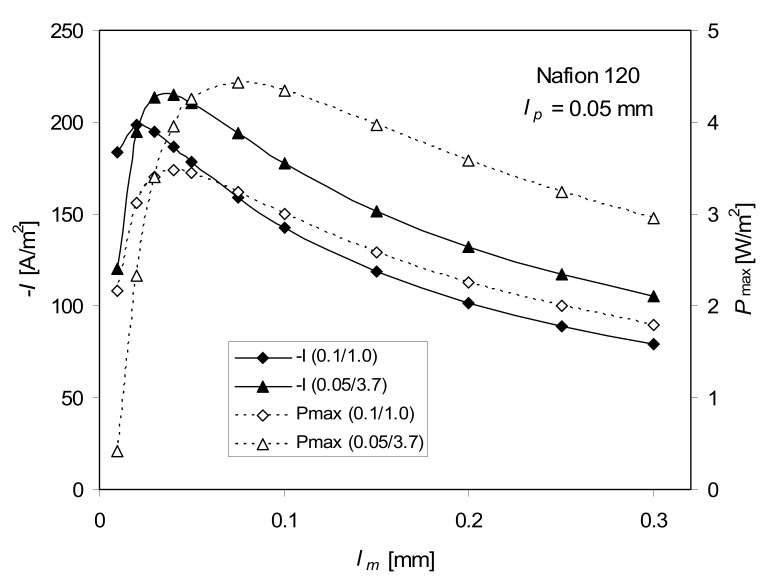
Dependence of the maximum of power density, *P*_max_, and the current density, −*I*, at *P*_max_ on the membrane thickness, *l_m_*, for 0.1–1.0 M and 0.05–3.7 M NaCl, *T* = 298 K, Equation (5), full ITE.

The influence of the membrane thickness on the maximum of *P* and the current density at *P*_max_ is shown in Figure 5. On both curves (*P*_max_ = *f*(*l_m_*) and −*I*(*P*_max_) = *f*(*l_m_*)), maxima are observed at *l_m_* < 0.1 mm, and the maximum on *P*_max_ is at a slightly higher *l_m_* value than that of −*I*(*P*_max_). The higher concentration difference demands a thicker membrane (maxima are shifted toward thicker membranes). The thinner the membrane, the higher *I*, but also the higher the permeability of electrolytes; the impact of diffusion layers (of the assumed constant thickness) also becomes relatively stronger.

**Figure 6:**
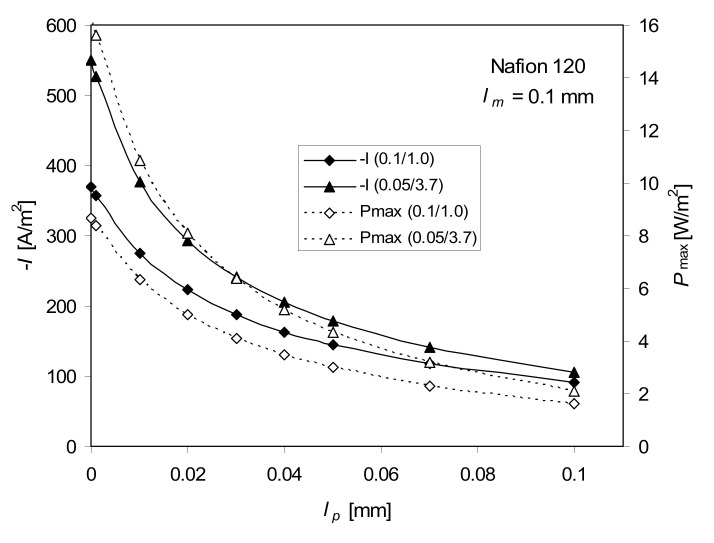
Dependence of the maximum of power density, *P*_max_, and the current density, *−I*, at *P*_max_ on the diffusion boundary layer thickness, *l_p_*, for 0.1–1.0 M and 0.05–3.7 M NaCl, *T* = 298 K, Equation (5), full ITE.

The dependence of the maximum of power density *P*_max_ and the current density at *P*_max_ on the diffusion boundary layer thickness *l_p_* is a monotonically decreasing function (Figure 6). Due to a substantial decrease in *P*_max_ with *l_p_*, the reduction of the thickness of that layer by ensuring adequate stirring of the solutions at the membrane surface is an important practical issue. Even more important is the decrease in *l_p_* on the lower concentration side of membrane. To reduce the concentration polarization effect, an extra energy for pumping is needed. 

### The Two-Coefficients Approach

**Figure 7:**
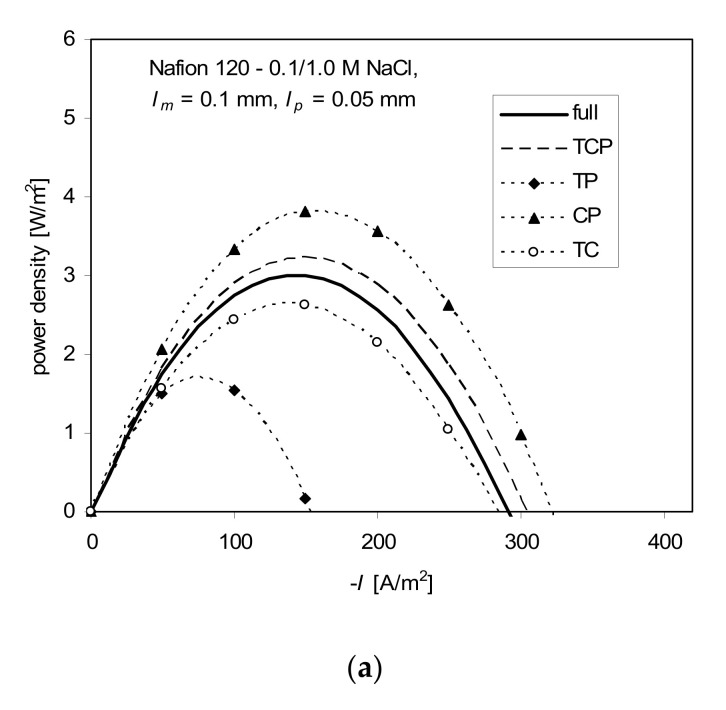

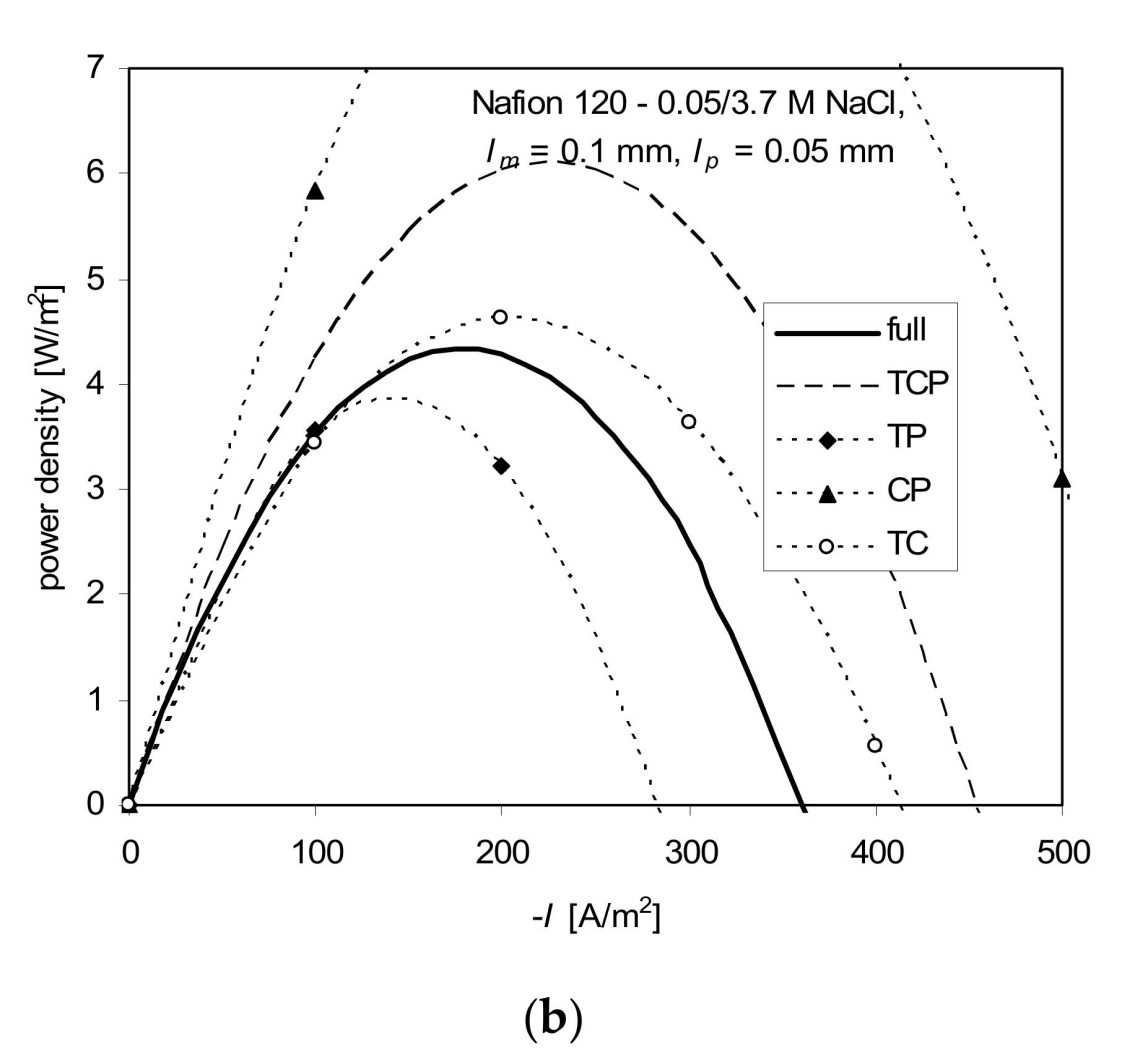
Power density vs. current density for full model and approximate approaches: TCP—3-coefficients approach (P_1_, P_2_, and L12 calculated from ${\bar{t}}_{1,app}$, *k_m_*, and *P*_s_), (t_1*app*_, *P_s_*); TP, CP, TC—2-coefficients approach (*P*_1_ and *P*_2_ calculated from ${\bar{t}}_{1,app}$ and *P*_s_, *k_m_* and *P_s_*, ${\bar{t}}_{1,app}$ and *k_m_*, respectively); the Nafion 120 membrane separates (**a**) 0.1 and 1 M NaCl, (**b**) 0.05 and 3.7 M NaCl; *l_m_* = 0.1 mm, *l_p_* = 0.05 mm, *T* = 298 K.

The results for the two-coefficients approach, for which *P*_1_ and *P*_2_ are calculated either from ${\bar{t}}_{1,app}$ and *k_m_* (TC), or from ${\bar{t}}_{1,app}$ and *P_s_* (TP), or from *k*_m_ and *P_s_* (CP), are shown in Figure 7. Among these three combinations of coefficients, the smallest deviation from the power density calculated from the full ITE was observed for the TC combination (${\bar{t}}_{1,app}$, *k_m_*). It yielded even better results than the three-coefficients approach based on all three parameters (TCP: ${\bar{t}}_{1,app}$, *k_m_* and *P_s_*).

Because of the quite good fit of the TC approach, another question arose: is it necessary to determine the concentration dependence of ${\bar{t}}_{1,app}$ and *k_m_* in the whole range *c*′–*c*″, or is it enough to determine ${\tilde{\bar{t}}}_{1,app}$ in that range and *k*_m_ for the mean concentration of *c*′ and *c*″?

In that case (*P*_1_, *P*_2_ = const), we can determine the integrated form for *J*_1_ from Equation (10):

(16) $$J_{1}=\frac{P_{1}}{P_{1}+P_{2}}j/F-\frac{P_{1}P_{2}}{\left(P_{1}+P_{2}\right)RTl_{m}}\int_{{c^{\prime }}_{s}}^{{c^{\prime \prime }}_{s}}\left(1-c_{s}{\bar{v}}_{s}\right)\frac{\partial \pi }{\partial c_{s}}dc_{s}.$$

Δ*E* can be expressed as a function of a certain mean concentration of *c′* and *c″*:

(17) $$\Delta E\equiv E^{\prime }-E^{\prime \prime }=\frac{RT}{F}\frac{\left(j/F-J_{1}\right)l_{m}}{{\tilde{c}}_{s}P_{2}}.$$

**Figure 8:**
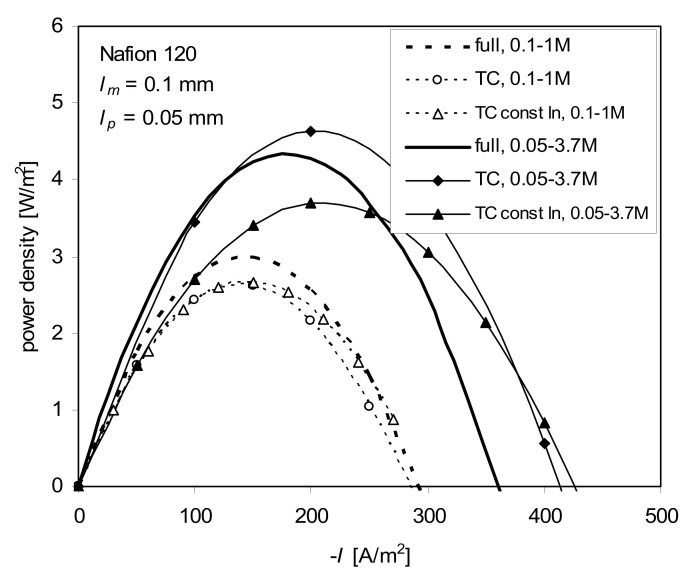
Power density vs. current density for full model—comparison of the TC const approach with logarithmic mean with the TC approach and the full ITE for the concentrations 0.1–1 M and 0.05–3.7 M NaCl; *l_m_* = 0.1 mm, *l_p_* = 0.05 mm, *T* = 298 K.

*P*_1_ and *P*_2_ are determined from *k*_m_ (Equation (12)), with *c*_s_ replaced by ${\tilde{c}}_{s}$, and ${\tilde{\bar{t}}}_{1,app}$ calculated from (15) with *dE*/*dμ*_s_ replaced by (*E*(*c*″) − *E*(*c*′))/(*μ*_s_(*c*″) − *μ*_s_(*c*′)). To check this simplification, the calculations were performed for ${\tilde{c}}_{s}$ being the arithmetic and logarithmic means of *c*′, *c*″. The boundary diffusion layers were treated as in the previous cases to keep the same conditions for all discussed approaches. To simplify calculations, in the calculations of *P*_1_, *P*_2_, and ${\tilde{c}}_{s}$, the external bulk concentrations were used, not those at the membrane surfaces. A comparison of this approach (TC const.) with the full ITE equation and with the TC approach is shown in Figure 8.

This simplification of the logarithmic mean of concentration (marked in Figure 8 as “TC const ln”) yields comparable results to the other approaches for the lower concentration difference (0.1–1.0 M). For 0.05–3.7 M the predictions of “TC const ln” are too low at the current density range 0–*j*(*P*_max_). 

In this case, the concentration dependence of ${\bar{t}}_{1,app}$ and *k_m_* is needed to improve the predictions of power density. The arithmetic mean of concentration yields much worse results especially for high concentration difference and is not shown in Figure 8.

## 4. Conclusions

Among the tested simplifications of the full set of transport equations of irreversible thermodynamics (six independent coefficients), the four-coefficients approach (*k_m_*, ${\bar{t}}_{1}$, ${\bar{t}}_{1,app}$, *P_s_*_,dif_) with only the pressure effect neglected yields almost the same results for 0.1–1 M as the full ITE approach.

The three-coefficients approach (*k_m_*, ${\bar{t}}_{1,app}$, *P_s_*_,dif_), where only ion fluxes are considered with their couplings, slightly overestimates the power density at lower concentration difference. However, at higher concentration differences, it strongly overestimates and becomes much less accurate than the two-coefficients approach (equation of the Nernst–Planck type, assuming no coupling between ion fluxes) based on *k_m_* and ${\bar{t}}_{1,app}$. Other combinations of the two-coefficients approach are substantially worse. The model based on *k_m_* and ${\bar{t}}_{1,app}$ can be further simplified by using constants *P*_1_ and *P*_2_ calculated from the mean values of *k_m_* and ${\bar{t}}_{1,app}$ in a given concentration range *c*′–*c*″ and using the logarithmic mean of these concentrations. These coefficients are easily accessible experimentally.

None of the discussed approximations considered the volume flow effect on the concentrations of the solutions contacting the membrane. To do so, we need to estimate the osmotic volume permeability and the electroosmotic coefficient *W*.

To obtain the optimal power density for given concentrations of feed solutions, the appropriate membrane thickness should be chosen. The higher the concentration difference, the thicker the membrane should be. As the position of *P*_max_ depends on the membrane properties, the optimal thickness should be independently determined for cation- and anion-exchange membranes.

The thickness of the diffusion boundary layer should be as low as possible especially on the low concentration side of the membrane—it strongly decreases the power density.

## Figures and Tables

**Table 1:** List of experiments, equation for 1:1 electrolyte.

Experiment	Equation
conductivity	$\kappa \equiv l_{m}{\left(\frac{I}{\Delta E}\right)}_{c,p}=l_{m}L_{EE}$
the real transport number of ion 1	${\bar{t}}_{1}\equiv {\left(\frac{FJ_{1}}{I}\right)}_{\Delta c,\Delta p=0}=\frac{L_{\pi E}}{L_{EE}}$
electroosmotic coefficient	$W\equiv {\left(\frac{FJ_{v}}{I}\right)}_{\Delta c,\Delta p=0}={\bar{v}}_{0}{\bar{t}}_{0}+{\bar{v}}_{s}{\bar{t}}_{1}=\frac{L_{pE}}{L_{EE}}$
the apparent transport number of ion 1 (electromotive force)	${\bar{t}}_{1,app}={\left(\frac{F\Delta E}{\Delta \mu_{s}}\right)}_{I,\Delta p=0}=\frac{F}{1-{\tilde{c}}_{s}{\bar{v}}_{s}}\left(\frac{L_{E\pi }}{L_{EE}}-{\tilde{c}}_{s}\frac{L_{Ep}}{L_{EE}}\right)$ ${\left({\bar{t}}_{1,app}\right)}_{\Delta c\to 0}={\bar{t}}_{1}-\frac{c_{s}}{c_{0}}{\bar{t}}_{0}$
electrolyte permeability	$L_{s}\equiv {\left(\frac{J_{s}}{\Delta \pi }\right)}_{I,\Delta p=0}=L_{\pi \pi }/{\tilde{c}}_{s}-L_{\pi p}+\frac{L_{Ep}L_{\pi E}-L_{\pi E}L_{E\pi }/{\tilde{c}}_{s}}{L_{EE}}$
osmotic permeability	$L_{v,osm}\equiv -{\left(\frac{J_{v}}{\Delta \pi }\right)}_{I,\Delta p=0}=L_{pp}-L_{\pi p}/{\tilde{c}}_{s}+\frac{L_{pE}L_{E\pi }/{\tilde{c}}_{s}-L_{pE}L_{Ep}}{L_{EE}}$
hydrodynamic permeability	$L_{p}\equiv {\left(\frac{J_{v}}{\Delta p}\right)}_{I,\Delta c=0}=L_{pp}-\frac{L_{pE}L_{Ep}}{L_{EE}}$

## Appendix A. Transport Equation in the Concentration Polarization Layers

In [39], the transport coefficients, *l_ik_*, are determined for the set of equations:

(A1) $$J_{i}^{\left(0\right)}=l_{i1}\left(-\frac{d{\tilde{\mu }}_{1}}{dx}\right)+l_{i2}\left(-\frac{d{\tilde{\mu }}_{2}}{dx}\right)\;i=1,\;2$$

in the solvent-fixed reference frame. Equation (A1) can be transformed with forces similar to those in Equation (3) (electrolyte 1:1):

(A2) $$\begin{array}{l} J_{1}^{\left(0\right)}=l_{11}a\left(-\frac{d\pi }{dx}\right)+l_{1E}\left(-\frac{dE}{dx}\right)\\ I=l_{1E}a\left(-\frac{d\pi }{dx}\right)+\kappa_{e}\left(-\frac{dE}{dx}\right) \end{array}$$

where $l_{1E}=F\left(l_{11}-l_{12}\right)$, $\kappa_{e}=F^{2}\left(l_{11}-2l_{12}+l_{22}\right)$, and $a=\left(1-c_{s}{\bar{v}}_{s}\right)/c_{s}$. Due to volume flow through the membrane, we assume that the flux of ion 1 in the boundary layer is given by:

(A3) $$J_{1}=J_{1}^{\left(0\right)}+\frac{c_{s}}{c_{0}}J_{0}.$$

In the practical equations (Equations (3) and (5)), *J_v_* is defined as $J_{v}={\bar{v}}_{0}J_{0}+{\bar{v}}_{s}J_{1}/\nu_{1}$; thus, for our case (electrolyte 1:1), Equation (A3) can be rewritten as:

$$J_{1}=J_{1}^{\left(0\right)}\left(1-c_{s}{\bar{v}}_{s}\right)+c_{s}J_{v}.$$

To simultaneously calculate the *c* and *E* profiles in the boundary layers with the membrane, we divide both layers into *n_e_* slices for which the transport equations are as follows:

(A4) $$\begin{array}{cc} J_{1}= & \left(l_{11}({\tilde{c}}_{s})a({\tilde{c}}_{s})\Delta \pi +L_{1E}({\tilde{c}}_{s})\Delta E\right)\left(1-{\tilde{c}}_{s}{\bar{v}}_{s}\right)/w_{sl,e}+{\tilde{c}}_{s}J_{v}\\ I= & \left(L_{1E}({\tilde{c}}_{s})a({\tilde{c}}_{s})\Delta \pi +\kappa_{e}({\tilde{c}}_{s})\Delta E\right)/w_{sl,e} \end{array}$$

where *w_sl,e_* is the width of the slices in those layers. Dividing each layer into *n_p_* slices we have (4*n_p_* + 3*n*_m_) equations (including equations for the membrane) and the same number of unknowns: (2*n_p_* + *n*_m_ − 1) of *c_s,k_*, (2*n_p_* + *n*_m_) of *E_k_*, (*n_m_* − 1) of *p_k_*, and *J*_1_, *J_v_*.

## References

[1] Lee K.H., Baker R.W., Lonsdale H.K. (1981). Membranes for power generation by pressure retarded osmosis. J. Membr. Sci..

[2] Levenspiel O., De Nevers N. (1974). The osmotic pump. Science.

[3] Narebska A., Koter S., Kujawski W. (1990). Conversion of Osmotic into Mechanical Energy in Systems with Charged Membranes. J. Non Equilib. Thermodyn..

[4] Kilsgaard B.S., Haldrup S., Catalano J., Bentien A. (2014). High figure of merit for electrokinetic energy conversion in Nafion membranes. J. Power Source.

[5] Manecke G. (1952). Membranakkumulator. Z. Physik. Chem..

[6] Pattle R.F. (1954). Production of Electric Power by mixing Fresh and Salt Water in the Hydroelectric Pile. Nature.

[7] Dlugolecki P., Gambier A., Nijmeijer K., Wessling M. (2009). Practical Potential of Reverse Electrodialysis As Process for Sustainable Energy Generation. Environ. Sci. Technol..

[8] Veerman J., de Jong R.M., Saakes M., Metz S.J., Harmsen G.J. (2009). Reverse electrodialysis: Comparison of six commercial membrane pairs on the thermodynamic efficiency and power density. J. Membr. Sci..

[9] Veerman J., Saakes M., Metz S.J., Harmsen G.J. (2010). Electrical Power from Sea and River Water by Reverse Electrodialysis: A First Step from the Laboratory to a Real Power Plant. Environ. Sci. Technol..

[10] Turek M., Bandura B. (2007). Renewable energy by reverse electrodialysis. Desalination.

[11] Giacalone F., Papapetrou M., Kosmadakis G., Tamburini A., Micale G., Cipollina A. (2019). Application of reverse electrodialysis to site-specific types of saline solutions: A techno-economic assessment. Energy.

[12] Veerman J., Post J.W., Saakes M., Metz S.J., Harmsen G.J. (2008). Reducing power losses caused by ionic shortcut currents in reverse electrodialysis stacks by a validated model. J. Membr. Sci..

[13] Veerman J., Saakes M., Metz S.J., Harmsen G.J. (2010). Reverse electrodialysis: Evaluation of suitable electrode systems. J. Appl. Electrochem..

[14] Guler E., Elizen R., Vermaas D.A., Saakes M., Nijmeijer K. (2013). Performance-determining membrane properties in reverse electrodialysis. J. Membr. Sci..

[15] Tedesco M., Hamelers H.V.M., Biesheuvel P.M. (2018). Nernst-Planck transport theory for (reverse) electrodialysis: III. Optimal membrane thickness for enhanced process performance. J. Membr. Sci..

[16] Vermaas D.A., Kunteng D., Saakes M., Nijmeijer K. (2013). Fouling in reverse electrodialysis under natural conditions. Water Res..

[17] Pintossi D., Saakes M., Borneman Z., Nijmeijer K. (2019). Electrochemical impedance spectroscopy of a reverse electrodialysis stack: A new approach to monitoring fouling and cleaning. J. Power Source.

[18] Vermaas D.A., Saakes M., Nijmeijer K. (2014). Enhanced mixing in the diffusive boundary layer for energy generation in reverse electrodialysis. J. Membr. Sci..

[19] Pawlowski S., Rijnaarts T., Saakes M., Nijmeijer K., Crespo J.G., Velizarov S. (2017). Improved fluid mixing and power density in reverse electrodialysis stacks with chevron-profiled membranes. J. Membr. Sci..

[20] Mehdizadeh S., Yasukawa M., Abo T., Kakihana Y., Higa M. (2019). Effect of spacer geometry on membrane and solution compartment resistances in reverse electrodialysis. J. Membr. Sci..

[21] Jeong H.I., Kim H.J., Kim D.K. (2014). Numerical analysis of transport phenomena in reverse electrodialysis for system design and optimization. Energy.

[22] Veerman J., Saakes M., Metz S.J., Harmsen G.J. (2011). Reverse electrodialysis: A validated process model for design and optimization. Chem. Eng. J..

[23] Kim D.K. (2011). Numerical study of power generation by reverse electrodialysis in ion-selective nanochannels. J. Mech. Sci. Technol..

[24] Tedesco M., Cipollina A., Tamburini A., van Baak W., Micale G. (2012). Modelling the Reverse ElectroDialysis process with seawater and concentrated brines. Desalin. Water Treat..

[25] Tedesco M., Cipollina A., Tamburini A., Bogle I.D.L., Micale G. (2015). A simulation tool for analysis and design of reverse electrodialysis using concentrated brines. Chem. Eng. Res. Des..

[26] Tedesco M., Hamelers H.V.M., Biesheuvel P.M. (2016). Nernst-Planck transport theory for (reverse) electrodialysis: I. Effect of co-ion transport through the membranes. J. Membr. Sci..

[27] Kim H., Jeong N., Yang S., Choi J., Lee M.S., Nam J.Y., Jwa E., Kim B., Ryu K.S., Choi Y.W. (2019). Nernst-Planck analysis of reverse-electrodialysis with the thin-composite pore-filling membranes and its upscaling potential. Water Res..

[28] Moya A.A. (2020). Uphill transport in improved reverse electrodialysis by removal of divalent cations in the dilute solution: A Nernst-Planck based study. J. Membr. Sci..

[29] Tedesco M., Hamelers H.V.M., Biesheuvel P.M. (2017). Nernst-Planck transport theory for (reverse) electrodialysis: II. Effect of water transport through ion-exchange membranes. J. Membr. Sci..

[30] Nikonenko V., Nebavsky A., Mareev S., Kovalenko A., Urtenov M., Pourcelly G. (2019). Modelling of Ion Transport in Electromembrane Systems: Impacts of Membrane Bulk and Surface Heterogeneity. Appl. Sci..

[31] Kedem O., Katchalsky A. (1963). Permeability of composite membranes. Part 1. Electric current, volume flow and flow of solute through membranes. Trans. Faraday Soc..

[32] Foley T., Klinowski J., Meares P. (1974). Differential Conductance Coefficients in a Cation-Exchange Membrane. Proc. R. Soc. Lond. Ser. A.

[33] Kumamoto E., Kimizuka H. (1981). Transport Properties of the Barium Form of a Poly(styrenesu1fonic acid) Cation-Exchange Membrane. J. Phys. Chem..

[34] Narebska A., Koter S., Kujawski W. (1985). Irreversible Thermodynamics of Transport across Charged Membranes. Part, I. Macroscopic Resistance Coefficients for a System with Nafion 120 Membrane. J. Membr. Sci..

[35] Narebska A., Kujawski W., Koter S. (1987). Irreversible Thermodynamics of Transport across Charged Membranes. Part II. Ion-Water Interactions at Permeation of Alkalis. J. Membr. Sci..

[36] McCallum C., Meares P. (1976). Computer prediction of stationary states of membranes from differential permeabilities. J. Membr. Sci..

[37] Krämer H., Meares P. (1969). Correlation of electrical and permeability properties of ion-selective membranes. Biophys. J..

[38] Koter S. (1993). Transport of electrolytes across cation-exchange membranes. Test of Onsager reciprocity in zero-current processes. J. Membr. Sci..

[39] Miller D.G. (1966). Application of Irreversible Thermodynamics to Electrolyte Solutions. I. J. Phys. Chem..

[40] Meares P. (1981). Coupling of ion and water fluxes in synthetic membranes. J. Membr. Sci..

[41] Dresner L. (1972). Stability of the Extended Nernst-Planck Equations in the Description of Hyperfiltration through Ion-Exchange Membranes. J. Phys. Chem..

